# Microscopic messengers: microbiota-derived bacterial extracellular vesicles in inflammatory bowel disease

**DOI:** 10.3389/fmicb.2024.1481496

**Published:** 2024-11-13

**Authors:** Muhammad Zubair, Fatma A. Abouelnazar, Ali Sobhy Dawood, Jingyun Pan, Xuwen Zheng, Tao Chen, Pengjun Liu, Fei Mao, Yongmin Yan, Ying Chu

**Affiliations:** ^1^Department of Laboratory Medicine, Wujin Hospital Affiliated With Jiangsu University, Changzhou, China; ^2^Wujin Institute of Molecular Diagnostics and Precision Cancer Medicine of Jiangsu University, Wujin Hospital Affiliated With Jiangsu University, Changzhou, China; ^3^Faculty of Applied Health Sciences Technology, Pharos University, Alexandria, Egypt; ^4^Medicine and Infectious Diseases Department, Faculty of Veterinary Medicine, University of Sadat City, Sadat, Egypt; ^5^Department of Traditional Chinese Medicine, Wujin Hospital Affiliated With Jiangsu University, Changzhou, China; ^6^Department of Emergency, Wujin Hospital Affiliated With Jiangsu University, Changzhou, China; ^7^Department of Gastroenterology, Wujin Hospital Affiliated With Jiangsu University, Changzhou, China; ^8^Key Laboratory of Medical Science and Laboratory Medicine of Jiangsu Province, Department of Laboratory Medicine, School of Medicine, Jiangsu University, Zhenjiang, Jiangsu, China; ^9^Wujin Clinical College, Xuzhou Medical University, Changzhou, China; ^10^Jiangsu Key Laboratory of New Drug Research and Clinical Pharmacy, Xuzhou Medical University, Xuzhou, China

**Keywords:** dysbiosis, extracellular vesicles, IBD, immune regulation, microbiota

## Abstract

Inflammatory bowel disease (IBD) is a persistent and complex condition accomplished by inflammation of the gastrointestinal system, encompassing Crohn’s disease (CD) and ulcerative colitis (UC). This condition is caused by the combination of genetic predispositions, environmental triggers, and dysregulated immunological responses, which complicates diagnosis and treatment. The latest developments in gastroenterology have revealed the critical significance of the gut microbiota in the pathogenesis of IBD. Extracellular vesicles (EVs) are a type of microbial component that potentially regulate intestinal inflammation. The impact of microbiota-derived bacterial EVs (bEVs) on intestinal inflammation is mediated through several methods. They can intensify inflammation or stimulate defensive responses by delivering immunomodulatory cargo. Improved comprehension could enhance inventive diagnostic and treatment strategies for IBD. This study aimed to explore the relationship between microbiota-derived bEVs and the complex nature of IBD. We performed a thorough analysis of the formation, composition, mechanisms of action, diagnostic possibilities, therapeutic implications, and future prospects of these microbiota-derived bEVs.

## 1 Introduction

IBD, encompassing UC and CD, involves chronic intestinal inflammation. While genetics play a role in IBD’s pathophysiology, the rapid rise in cases suggests that environmental factors are also crucial (Matsuoka and Kanai, 2015). UC commonly causes colon inflammation, and symptoms include abdominal cramps, bloody diarrhea, bowel incontinence, and hematochezia. Inflammation is mainly restricted to the mucosal and submucosal layers (Porter et al., 2020). In CD, inflammation can lead to penetrating fistulas or fibrostenotic obstructions, with fistulas occurring in 20–40% of patients. Fistulas can form between the bowel and adjacent structures such as the bladder, skin, or vagina (Schwartz et al., 2002). UC is a chronic disease manifested by diffuse inflammation of the rectal and colonic mucosa, involving the rectum in 95% of cases and potentially extending to the proximal large intestine. The classic symptom is bloody diarrhea. The disease course includes periods of remission and exacerbation, occurring spontaneously or with treatment (Da Silva et al., 2014). Inflammation is limited to the colonic mucosa, resulting in symptoms that are less varied than those of CD. Symptoms, including diarrhea, tenesmus, and abdominal pain, vary with the extent of inflammation. Systemic symptoms such as fatigue, fever, and weight loss may also occur (Flynn and Eisenstein, 2019). The majority of the patients with UC experience bloody diarrhea, with bleeding severity linked to the extent of colonic involvement. Those with distal disease may pass only bloodstained mucus or small amounts of fresh blood (Ordás et al., 2012).

The incidence of pediatric onset of IBD, mainly CD, appears to be increasing for unknown reasons. This trend has been observed in Western regions such as Canada, France, and northern Europe and in former eastern European countries such as the Czech Republic, Croatia, and Hungary (Benchimol et al., 2011). Nearly 1.6 million Americans have IBD, with 785,000 affected by CD and 910,000 by UC. Both conditions usually onset in the 2nd or 4th decade of life, with CD typically appearing between the ages of 15 and 25 years and UC between the ages of 25 and 35 years, without a gender preference (Molodecky et al., 2012). In the past decade, the incidence of IBD has risen among Asian and Hispanic populations. Moreover, individuals who migrate from low-prevalence areas to high-prevalence areas, especially their children born in these new regions, face a higher risk of developing IBD (Flynn and Eisenstein, 2019). A recent systematic review study disclosed the incidence of IBD was established in the Western world since 1990, though prevalence remained high. Newly industrialized countries face increasing incidence rates, similar to past Western trends (Ng et al., 2017).

Despite numerous studies published elaborating the pathogenesis of IBD, it is still poorly understood. Several factors are involved, which make the understanding of IBD pathogenesis challenging. These include complex genetics (Luke, 2012), environmental factors (Kaplan, 2015), immune system dysregulation (Neurath, 2014), epigenetic factors (Ventham et al., 2016), microbiota interaction (Curtis and Kostic, 2014), limitations of animal models to successfully replicate in humans (Atsushi, 2012), and disease variability among the patients (Cleynen and Vermeire, 2015). Limited knowledge about its pathogenesis hinders the development of diagnostic and therapeutic strategies. It is imperative to study the role of various factors, especially microbiota, in the pathogenesis of IBD.

The human microbiome comprises various types of microbes that colonize distinct niches within the human body, including the skin, lungs, vagina, and gastrointestinal tract (GIT). Among these, the gut microbiota is the most extensively studied due to its greater diversity and higher number of microbial species compared to other bodily regions (Chloé and Sokol, 2020; Quigley, 2013). The gut microbiota is a complex community of approximately 100 trillion microorganisms, including bacteria, fungi, viruses, and protists (Quigley, 2013). Recent research has highlighted the key role of microbiota-derived EVs in the pathophysiology of IBD. These nano-sized vesicles, secreted by gut microbes, can transport a diverse array of biomolecules comprising lipids, proteins, and nucleic acids. This capability allows microbiota-derived EVs to influence host immune responses and maintain gut homeostasis (Wang et al., 2023). These play a significant role mainly in modulating the inflammatory pathways, maintaining gut barrier integrity (Chelakkot et al., 2018), communication (Buzas et al., 2014), and delivering drugs (Yang et al., 2018).

## 2 Unraveling the microbial messengers: microbiota EVs

Microbiota-derived EVs are small, membrane-bound particles released by microorganisms, including bacteria, fungi, and archaea within the microbiota. The biomolecules harbored by these vesicles facilitate communication between microbes and their hosts, as well as interactions among microbial communities (Kulp and Kuehn, 2012). Due to the abundant bacterial population in the microbiota and their significance, we will focus on bEVs. Vesicles derived from Gram-negative bacteria originate from the outer membrane (enveloped with a double periplasmic layer) and are regarded as outer membrane vesicles (OMVs), while vesicles derived from Gram-positive bacteria (enveloped with a peptidoglycan layer) originate from the cytoplasm and are termed as cytoplasmic membrane vesicles (CMVs) (Toyofuku et al., 2019; Schwechheimer and Kuehn, 2015; Kim et al., 2015). Bacteria also release exosomes that are smaller in size (30–150 nm) than exosomes released by OMVs (Niel, Van Niel et al., 2018). Bacterial vesicles usually vary in shape and composition depending on strain, growth conditions, and route of biogenesis. Biochemical and proteomic analysis revealed that EVs are mainly composed of proteins, lipids (phospholipids and lipopolysaccharides), nucleic acids (DNA and RNA), enzymes, and toxins (Van Niel et al., 2018; Quaglia et al., 2020).

### 2.1 Biogenesis of microbiota EVs

There are different ways of biogenesis of membrane vesicles from Gram-negative and Gram-positive bacteria. Various models have been proposed to describe the biogenesis of OMVs from Gram-negative bacteria (Volgers et al., 2018). Model one describes the generation of vesicles as a result of defects in the irregular distribution of lipids with a lipopolysaccharide layer at the outer side and phospholipids at the inner side of the membrane (Malinverni and Silhavy, 2009). Another aspect of this model is the instability in the bacterial envelope due to the disruption of crosslinks between peptidoglycan and outer membrane bridged via Braun’s lipoproteins that are responsible for cell wall integrity (Deatherage et al., 2009). The second model describes the accumulation of misfolded proteins or fragments of peptidoglycan exerting pressure on the outer membrane, resulting in the generation of vesicles (Wessel et al., 2013). This phenomenon can be provoked by temperature stress or deformity in the outer membrane. The third model involves the induction of membrane curvature by the bacteria that is finally shaped into the cell envelope. This model finds its best example in *P. aeruginosa* in which the signaling molecule pseudomonas quinolones assists its packaging and transmission by inducing membrane curvature and formation of OMVs (Schertzer and Whiteley, 2012). Vesicles in Gram-positive bacteria are produced mainly due to cell lysis such as endolysin generates holes in the peptidoglycan cell wall. Cytoplasmic membrane material protrudes into the extracellular area through these holes and is discharged as membrane vesicles. Other ways include turgor pressure and bubbling death, as reviewed previously (Toyofuku et al., 2017). *Staphylococcus aureus* also exhibits this phenomenon for vesiculation (Andreoni et al., 2018).

### 2.2 Microbiota bEVs cargo

bEVs are mainly composed of proteins, lipids, nucleic acids, and cytoplasmic components. These also encompass various cell surface constituents, including outer membrane proteins (OMPs), peptidoglycan (PG), and lipopolysaccharides (LPS), and may also serve as transporters for diverse bacterial components such as nucleic acids, enzymes, toxins, and a complex of microbe-associated molecular patterns (MAMPs) (Filip, 2021). The size of bEVs and the nature of the contents of secreted molecules from the bacteria and their packaging largely depend on the particular species (Zakharzhevskaya et al., 2017), environment (Nicole and Meta, 2017; Stentz et al., 2022), antibiotics (Andreoni et al., 2018), protein localization (McMahon et al., 2012), and genetics (Vasilyeva et al., 2009). The size of EVs originating from Gram-negative bacteria ranges from 10 nm to 300 nm, while the size of EVs originating from Gram-positive bacteria ranges from 20 nm to 400 nm. EVs derived from Gram-negative bacteria are mainly composed of OMPs, periplasmic proteins, virulence factors, cytoplasmic proteins, inner membrane proteins, LPS, phospholipids, and peptidoglycan (10–20%). Unlike Gram-negative bacteria, EVs of Gram-positive bacteria are composed of cytoplasmic proteins, membrane-associated proteins, peptidoglycan (>50%), and lipoteichoic acid (LTA) (Yu et al., 2018; Tian et al., 2023). Microbiota-derived bEVs from beneficial or harmful bacteria affect intestinal and immune barriers; hence, they are involved in the mitigation or progression of the inflammatory process in IBD, respectively. Upon internalization in human intestinal epithelial cells, Gram-negative bacterial OMVs release LPS into the cytosol, a process mediated by sorting nexin 10 (SNX10), which activates caspase-5. This results in Lyn phosphorylation, which subsequently downregulates E-cadherin production and damages the intestinal barrier (Wang et al., 2021). Other bacterial species in the microbiota secrete specific molecules that regulate gut homeostasis and intestinal barrier integrity (Table 1).

**Table 1:** Role of specific molecules harbored by bEVs in IBD progression.

Bacteria	Specific secreted molecules	Functions	Reference
EHEC O157	1-Hemolysin 2-CdtV-B	1-Increases mitochondrial permeability and triggers apoptosis 2-DNA damage leading to apoptosis	Bielaszewska et al. (2013, 2017)
*Campylobacter jejuni*	Cytolethal distending toxins (CDT)	Damage cellular DNA	Lindmark et al. (2009)
*V. cholerae*	Active proteases	Induce apoptosis or necrosis	Mondal et al. (2016)
Enterotoxigenic *B. fragilis*	*B. fragilis* toxins	Disrupt the intestinal barrier via cleaving E-cadherin	Zakharzhevskaya et al. (2017)
*Fusobacterium nucleatum*	FomA	Gut immunomodulation through NF-kB response	Martin-Gallausiaux et al. (2020)
*S. aureus*	Lipoproteins	Initiation of pro-inflammatory response	Kang et al. (2015)
*Bacteroides thetaiotaomicron*	Anaerobic sulfatase maturating enzyme (anSME)	stimulate colitis in dnKO mice	Hickey et al. (2016)
Adherent invasive *E. coli* (AIEC) LF82	OmpA and OmpC	Exacerbate inflammation and IBD progression	Rolhion et al. (2005)

## 3 Role of microbiota EVs in intercellular communication and protection of gut health

bEVs are integral to intercellular communication within the gut microbiota, mediating various processes that contribute to microbial ecology, host interactions, and overall gut health. Their ability to transfer a diverse array of molecules enables them to influence bacterial behavior, host immunity, gut barrier integrity, and gene transfer, highlighting their multifaceted role in the gut ecosystem.

**Figure 1:**
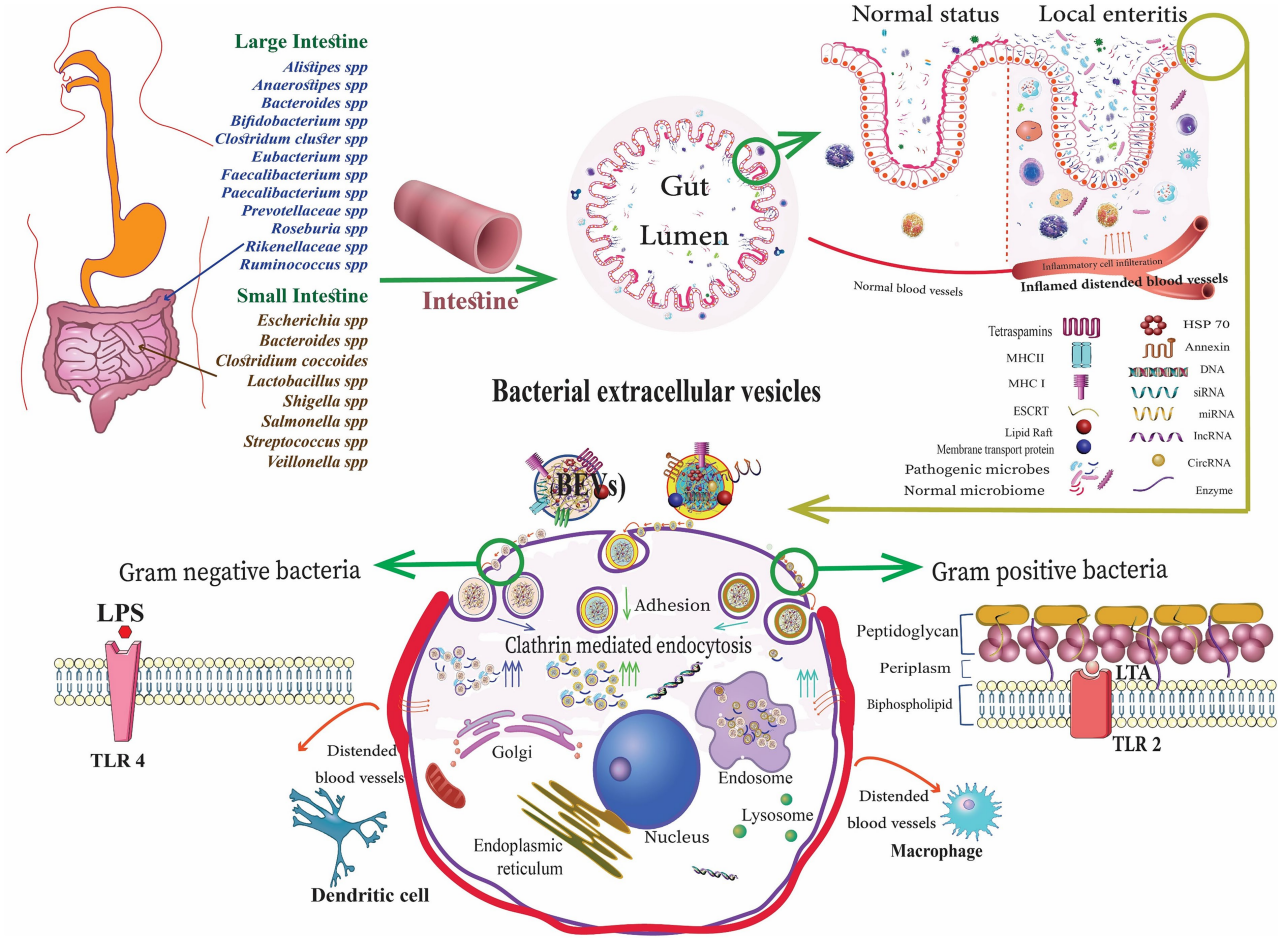
Gut microbiota-derived bacterial extracellular vesicles (bEVs) entry into the host cell after binding with the cell receptors, delivering the content, and initiating the cellular response (Alberti et al., 2021).

bEVs released by commensal bacteria and probiotics can relieve IBD by influencing the human immune system and intestinal barrier in the intestinal tract (Table 2). These bEVs either contribute to the maintenance of homeostasis or can lead to the development of diseases. The processes by which host cells take up bEVs involve interactions with host receptors, delivery of cargo, and complete integration into the cytoplasm of the host cell. The routes of uptake include endocytosis, phagocytosis, and direct membrane fusion (Ahlawat et al., 2021). Toll-like receptors (TLRs) and NOD-like receptors (NLRs) play a critical role in detecting bEVs. Gram-negative bacteria possess LPS and are known to engage TLR4, while Gram-positive bacteria are enriched with LTA and engage TLR2 for the uptake of EVs (Figure 1). The EVs derived from probiotic bacteria such as *Bifidobacterium* and *Lactobacillus* improve immune responses (Stange and Schroeder, 2019; Hao et al., 2021). These can improve the non-specific cellular immune response, which is marked by the activation of macrophages, NK cells, antigen-specific cytotoxic T lymphocytes, and the release of various cytokines, in a manner that is both strain-specific and dose-dependent (Ashraf and Shah, 2014). In addition, cellular LPS-binding proteins aid in capturing bEVs that expose LPS.

**Table 2:** Bacterial EVs (bEVs) derived from beneficial bacteria play a role in immunomodulation.

Origin	Methods	Findings/Function	Ref.
*A. muciniphila*	Metagenomic analysis	Enhanced the synthesis of IL-6, a pro-inflammatory cytokine, in colon epithelial cells stimulated by *E. coli* EVs	Kang et al. (2013)
*L. casei*	Proteomic approach (LC–MS)	Secretory proteins, heat and cold shock proteins, metabolic enzymes, proteases, membrane transporters, hydrolases, P40, P75, and LCABL-31160. Playing role in adhesion and subsequent inflammatory responses	Domínguez Rubio et al. (2017)
*Escherichia coli* (EHEC) O157	Purification of OMVs and cytokine production assay	Stimulate the production of IL-8 in IECs via the TLR5 and TLR4/MD-2 complex signaling pathway, followed by the activation of NF-_B	Bauwens et al. (2018)
*Bacteroides fragilis*	Quantitative reverse transcriptase PCR and cytokine concentration assay	Capsular polysaccharide (PSA) derived from OMVs may exert immunomodulatory effects and prevent mucosal inflammation	Ahmadi Badi et al. (2019)
*Escherichia coli* Nissle 1917 and ECOR12	Time course measurement of fluorescence and microscopy analysis	Activate NOD1 signaling pathways in IECs EV-mediated activation of NF-_B and further expression of IL-6 and IL-8	Cañas et al. (2016)
*Bacteroides thetaiotaomicron*	Single-cell RNA sequencing data, host-microbe p–p interaction, and TLR pathway analysis	Hydrolytic enzyme-containing EVs, which increase the digestion of gut microbiota, and they can share them with bacteria lacking these hydrolytic enzymes	Gul et al. (2022)
*Bacteroides acidifaciens*	Proteomics analysis	Protective effect on colitis, including alleviating the colitis phenotype, reducing inflammatory response, and improving intestinal barrier function	Zheng et al. (2023)

*Pediococcus pentosaceus* is a species of bacteria that produces lactic acid and can reduce inflammation. This bacterium releases EVs that stimulate the transformation of macrophages into an anti-inflammatory M2 phenotype by interacting with TLR2 receptors (Alpdundar Bulut et al., 2020). The probiotic *E. coli* Nissle 1917 (EcN-1917) EVs stimulate Dendritic cells (DCs) to initiate a Th1 immune response, essential for defending against pathogens (Hu et al., 2020). Polysaccharides transported by *Bacteroides fragilis*-derived bEVs engage with TLR2 receptors on DCs, leading to the stimulation of regulatory T cells (Tregs), promoting the production of anti-inflammatory cytokines (Ahmadi Badi et al., 2019). The EVs of *Propionibacterium freudenreichii*, which contain the surface-layer protein B (SlpB), can prevent the release of IL-8 caused by LPS (Mantel et al., 2024). This demonstrates the importance of the protein cargo in modulating the immune response.

bEVs can relieve intestinal inflammation by decreasing endoplasmic reticulum stress. For instance, the application of EVs derived from *Lactobacillus paracasei* to HT29 cells activated with LPS and mice with colitis induced by Dextran sulfate sodium (DSS) resulted in a significant reduction in inflammatory markers and improvement in colitis symptoms (Huang et al., 2021).

Multiple research studies on gut microbiota species have revealed the involvement of bEVs in regulating the integrity of the epithelial barrier (Martínez-López et al., 2019; Kayama and Takeda, 2020; Kayama et al., 2020; Wu et al., 2020; Barbara et al., 2021; Di Tommaso et al., 2021; Gieryńska et al., 2022). Regarding intestinal *E. coli* isolates, the probiotic EcN-1917 has undergone thorough investigation due to its well-established immunomodulatory and anti-inflammatory capabilities that effectively promote microbiota equilibrium (Sonnenborn et al., 2016). The probiotic EcN-1917 strengthens the barrier of epithelium by controlling the expression of tight junction proteins, that is, ZO-1, ZO-2, claudin-14 (Babickova and Gardlik, 2015), and the desmosome protein pinin (Qiao et al., 2021). Gao et al. demonstrated that the probiotic component responsible for increasing the expression of claudin-14 is the secreted protein TcpC. TcpC activates the ERK1/2 signaling pathway (Gao et al., 2022). Further research conducted using EcN-1917 cell-free supernatants has validated that secreted bEVs, in addition to TcpC, possess the ability to enhance barrier function (Zhang et al., 2024).

EcN-1917 and ECOR63 strain-derived EVs upregulate tight junction proteins, including ZO-1 and claudin-14, while decreasing the claudin-2 expression. This leads to an improvement in the barrier function and a decrease in permeability (Alvarez et al., 2019). The cargo of gut microbiota-derived bEVs is primarily responsible for the beneficial impact they have on IBD. *Lactobacillus reuteri* EVs contain nucleic acids and bioactive proteins such as glucosyltransferase, serine protease, and elongation factor Tu. These proteins can regulate immunological responses. EVs administered to broilers challenged with LPS decreased inflammation, enhanced survival, and regulated the expression of pro- and anti-inflammatory genes in the jejunum (Hu et al., 2021).

## 4 Dysbiosis and the gut microbiota-derived bEVs axis in inflammatory bowel disease

### 4.1 Dysbiosis and altered microbiota composition in IBD pathogenesis

IBD is marked by dysbiosis, characterized by a decrease in the overall number and variety of species. Several presentations of dysbiosis have certain shared biological and microbiological characteristics (Hrncir, 2022; Haneishi et al., 2023). The effects observed include (a) a decrease in bacteria that produce short-chain fatty acids (SCFAs), specifically *Faecalibacterium*, *Roseburia*, *Lachnospiraceae*, and *Eubacterium*, which are essential for butyrate production; (b) a disruption of the beneficial symbiont *Akkermansia muciniphila*, which has anti-inflammatory properties; (c) a decrease in hydrogen and methane production in the intestine, accompanied by an increase in hydrogen sulfide (H_2_S), which is harmful to the intestinal lining; (d) an increase in the abundance of Gram-negative bacteria, particularly *Proteobacteria*, that express LPS in their outer membrane, also known as endotoxin, which contributes to inflammation; and (e) an increase in oxidative stress in the mucosa, leading to bacterial growth in areas with higher oxygen availability (Drago et al., 2019).

**Figure 2:**
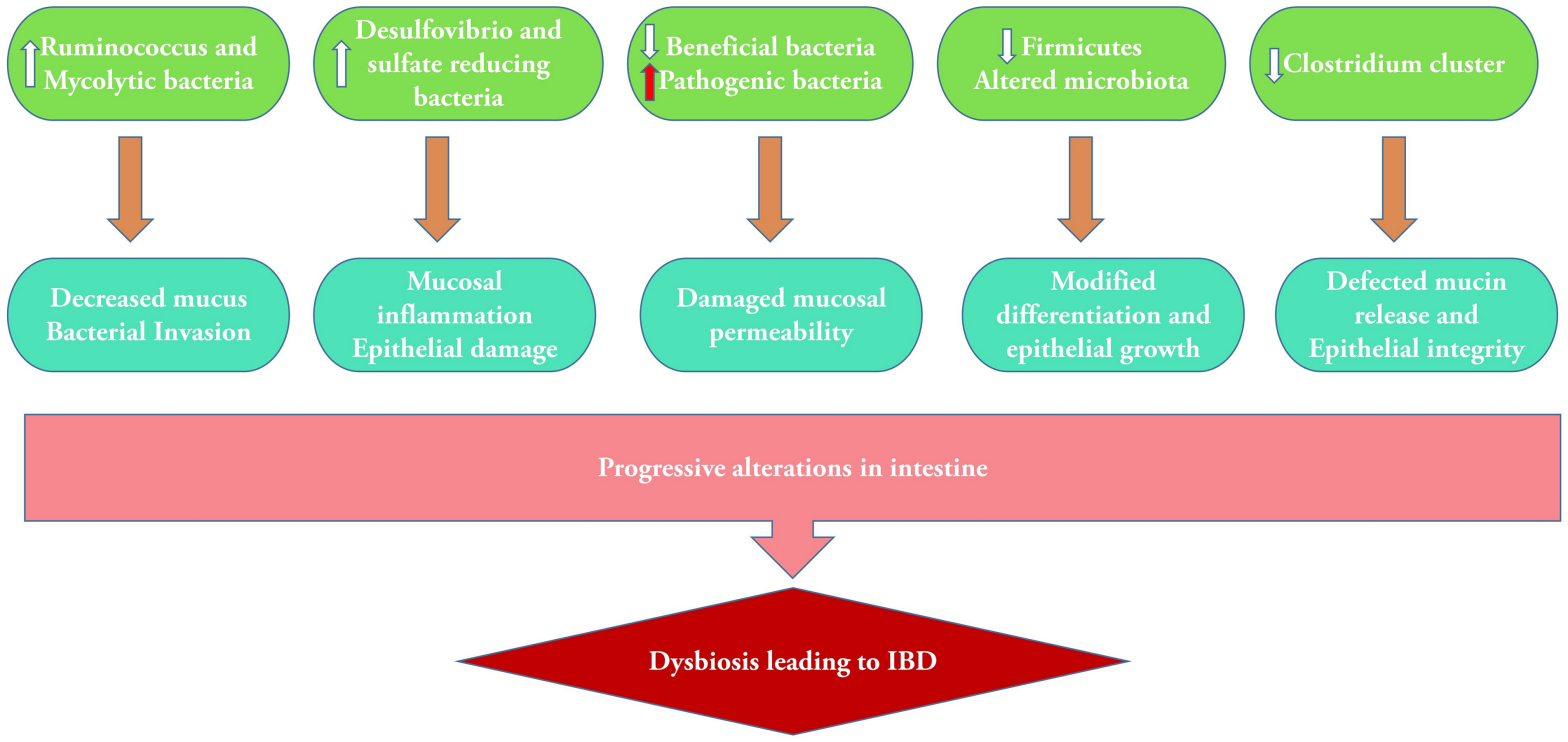
Alterations in microbiota containing bacterial population in dysbiosis and subsequent mucosal damage leading to IBD.

The metagenomics technique revealed a decreased level of diversity in the bacterial phylum Firmicutes, which serves as a characteristic feature of the fecal microbiota in individuals with CD (Manichanh et al., 2006). The abundance and richness of *C. leptum* were considerably decreased in both CD and UC (Kabeerdoss et al., 2013). The most severe gut dysbiosis is observed during *Clostridium difficile* infection, characterized by a significant proliferation of a single “keystone pathogen,” *C. difficile*. This can potentially lead to the development of a life-threatening IBD known as pseudomembranous colitis. Metagenomics study revealed that microbiota functions were more disturbed with 15% changes in pathways than 2% changes in microbiota composition. Major changes were noticed in oxidative stress pathways, as well as reduction in carbohydrate metabolism and amino acid biosynthesis (Morgan, 2012). The primary factors contributing to gut dysbiosis are the reduction of beneficial bacteria, the proliferation of pathobionts, and the decrease in microbial diversity (Buttó and Haller, 2016; Figure 2). The dysbiosis associated with IBD causes a decrease in the activity of microbial enzymes, leading to variations in the composition of bile acids in the gut. Modified bile acid transformation in the gastrointestinal lumen can nullify the anti-inflammatory properties of certain bile acid species on the gut epithelium, perhaps contributing to the ongoing cycle of chronic inflammation in IBD (Duboc et al., 2013). A study has shown a clear link between an imbalance in the salivary microbiota and IBD. It was validated that specific bacteria, such as *Prevotella* and *Veillonella*, were highly abundant in individuals with this disease. A notable rise was observed in the levels of the harmful bacteria *Clostridium sensu stricto* 1 and *Escherichia–Shigella* in the fecal samples of individuals with IBD. This increase was accompanied by a considerable enhancement of metabolic pathways related to bacterial invasion, suggesting damage to the intestinal epithelium (Abdelbary et al., 2022).

Microbiota dysbiosis is more distinct in patients with CD than in patients with UC (Pascal, 2017). Furthermore, the stability and diversity of the microbial community are much lower in CD than in UC (Jung et al., 2019). In addition, it is essential to highlight that the collection of fecal and mucosal samples is crucial for assessing microbial dysbiosis in IBD (Richard et al., 2018). In individuals with IBD, there is a notable reduction in the growth of *Lactobacillus*, *Ruminococcus*, *Bifidobacterium,* and *Clostridia* with an increase in the growth of *Veillonellaceae*, *Pasteurellaceae*, *Enterobacteriaceae*, and *Fusobacteriaceae*, compared to the healthy population (Kostic et al., 2014). *Lactobacillus* is recognized for its ability to restore the changed proportions of immune cells, as demonstrated in numerous studies, which highlighted its enhanced anti-inflammatory capacity and inhibition of pro-inflammatory activity. *Lactobacillus reuteri* not only inhibited the recruitment of neutrophils and the proliferation of DCs in the intestinal mucosa but also augmented the prevalence of Tregs in the mesenteric lymph nodes (Hoang et al., 2018). *Lactobacillus rhamnosus* effectively modulated the relative quantity of immune cells by reducing the Th17/Treg ratio via the JAK–STAT signaling pathway, facilitated by TLR2, in the colons of mice with DSS-induced colitis (Tong et al., 2021). *Lactobacillus plantarum*-derived EVs significantly reduced the levels of pro-inflammatory cytokines, including IL-6, IL-1β, IL-2, and TNF-*α*. A reduction in Proteobacteria and an increase in anti-inflammatory bacteria including *Bifidobacteria* and *Muribaculaceae* were also observed (Hao et al., 2021).

### 4.2 Dysregulated microbiota EVs profiles in IBD patients

The aspect of dysregulated microbiota EV profile has not been studied extensively. However, a recent study demonstrated a notable disparity in the makeup of the microbiota comprising bacteria and their MVs in patients with CD and healthy controls. Ultrafiltration and size exclusion chromatography were used to obtain fecal bacterial membrane vesicles (fbMVs). DNA was extracted from the fbMV fraction, which refers to the pellet recovered from dissolved feces containing bacterial DNA (bDNA) or straight from feces regarded as fecal DNA (fDNA). Beta-diversity analysis revealed a notable dissimilarity between the microbial community structure of the fMVs and the microbial profiles of the fDNA and bDNA. The diversity of bacteria found in the membrane vesicles of fecal samples was dramatically reduced in individuals with CD compared to healthy controls and even lower in those with active disease. The analysis of fDNA and bDNA revealed that *Firmicutes* were most abundant. However, in fbMVs, *Bacteroidetes* was the leading phylum. The composition of *Firmicutes* and *Proteobacteria* families and genera in fMVs was differed considerably between individuals with CD and healthy controls (Kameli et al., 2021). It is evident that the EV profile cannot indicate the actual bacterial type or the population in the gut during IBD. However, altered gut microbiota can eventually modify the EV profile, including EVs derived from some pathobionts playing their role in subsequent inflammation. Upon the administration of gut pathobiont-derived OMVs to Mdr2−/− mice, a notable increase in liver inflammation and fibrosis was noticed. It was confirmed that the existence of OMVs in the bloodstream and liver areas affects severe fibrosis using a group of patients with primary sclerosing cholangitis and IBD (Dorner et al., 2024).

### 4.3 Dysbiotic microbiota EVs and their role in the progression of IBD

bEVs have been extensively investigated in several pathological circumstances, including colorectal cancer (CRC) and IBD. As mentioned in Section 3, bEVs play a protective role in IBD by improving gut integrity and health. However, these are also becoming increasingly recognized as factors that cause inflammation and immune system dysfunction, mainly due to a dysbiotic microenvironment, leading to damage to the GIT. The increased prevalence of Gram-negative bacteria observed in patients with IBD generally results in an abundance of OMVs rich in LPS. These OMVs penetrate epithelial cells, and their LPS translocates into the cytosol, provoking immunological responses, downregulating E-cadherin expression, and inducing intestinal barrier failure (Wang et al., 2021). The role of heat shock protein (Hsp), namely Hsp70, in developing IBD has been studied. Hsp70 is mainly abundant in EVs. Exosomal HSP70 binds to receptors found in Gram-negative bacteria (TLR4) and Gram-positive bacteria (TLR2), which triggers pro-inflammatory reactions. In addition, exosomal HSP72 has a role in the functions of intestinal epithelial cells (IECs) (Samborski and Grzymisławski, 2015). EVs may have a pro-inflammatory effect in active IBD by stimulating, sustaining, and controlling the necessary functions of intestinal tissues. The modulatory properties demonstrated by EVs make them highly suitable candidates for the management and prevention of IBD (Bavisotto and Fais, 2017). At present, the excessive activation of pathways that promote inflammation is prevented by inhibiting certain biomolecules such as tumor necrosis factor TNF-*α*, gut-homing α4β7 integrin, and IL-12 (Hindryckx et al., 2018). This is done because bacteria trigger various pathways that might include these biomolecules as mediators to cause harmful effects, such as infections and malignancies (Bonovas et al., 2016). Enteropathogenic bacteria-derived EVs stimulate the release of EVs from the intestinal mucosa. These EVs include a high concentration of C–C motif chemokine 20 (CCL20) and prostaglandin E2 (PGE2), leading to inflammation.

Furthermore, it has been noted that toxins can infect EVs, which can then be discharged by certain intestinal bacteria, fostering the progression of CRC by intensifying an inflammatory state. For instance, *Bacteroides fragilis* secretes fragilis toxin into EVs, which can stimulate the formation of colon tumors by causing the breakdown of E-cadherin and the release of IL-8 (Bavisotto and Fais, 2017). Patients with CRC experience an elevation in the abundance of *Bacteroidetes*. These bacteria manage inflammation by regulating the development of Tregs. The immunoregulatory features of capsular polysaccharide A, produced by *B. fragilis*, involve transforming CD4+ T cells to Foxp3+ Treg through TLR2-mediated signaling. Such cells exhibit a higher ability to suppress immune responses due to their increased production of the anti-inflammatory cytokine interleukin (IL-10) (Round and Mazmanian, 2009).

## 5 Therapeutic applications and future perspectives

Fecal microbiota transplantation (FMT) is a widely used treatment that restores the normal microbiome by reconstituting gut flora. Although FMT demonstrates a high efficacy rate of 85–90%, it is associated with significant adverse effects, which may include minor symptoms such as diarrhea and bloating, as well as rare but severe consequences such as infections, gastrointestinal perforations, and mortality in high-risk individuals. Individuals with impaired immune systems and those suffering from IBD face an elevated risk, including flares or infections (Dailey et al., 2019). The utilization of biological medications is another therapeutic approach for IBD. A study investigated adverse events (AEs) and therapeutic failures linked to biological medications for treating IBDs in Southern Italy from 2019 to 2021. A total of 358 patients received adalimumab, golimumab, vedolizumab, ustekinumab, and infliximab (the most commonly used medication). Adverse events occurred in 20.4% of patients, while 17.3% encountered treatment ineffectiveness (Tallarico et al., 2022). Stojanov and Berlec reviewed the utilization of smart bio-nanomaterials including smart hydrogels (three-dimensional networks of hydrophilic polymers that can absorb water and retain significant quantities of it while preserving their structure through chemical or physical cross-linking of individual polymer chains), smart nanoparticles (polymeric material with dimensions less than 100 nm), and smart nanofibers (polymeric fibrous materials with nanometer diameters, high surface-to-volume ratios, low weight, and high, controllable porosity) in IBD therapy. These nano-biomaterials are capable of delivering the active ingredients to the target area in a controlled manner but have disadvantages such as difficulty in loading the hydrophobic drugs, stability, and an expensive process (Stojanov and Berlec, 2024).

**Figure 3:**
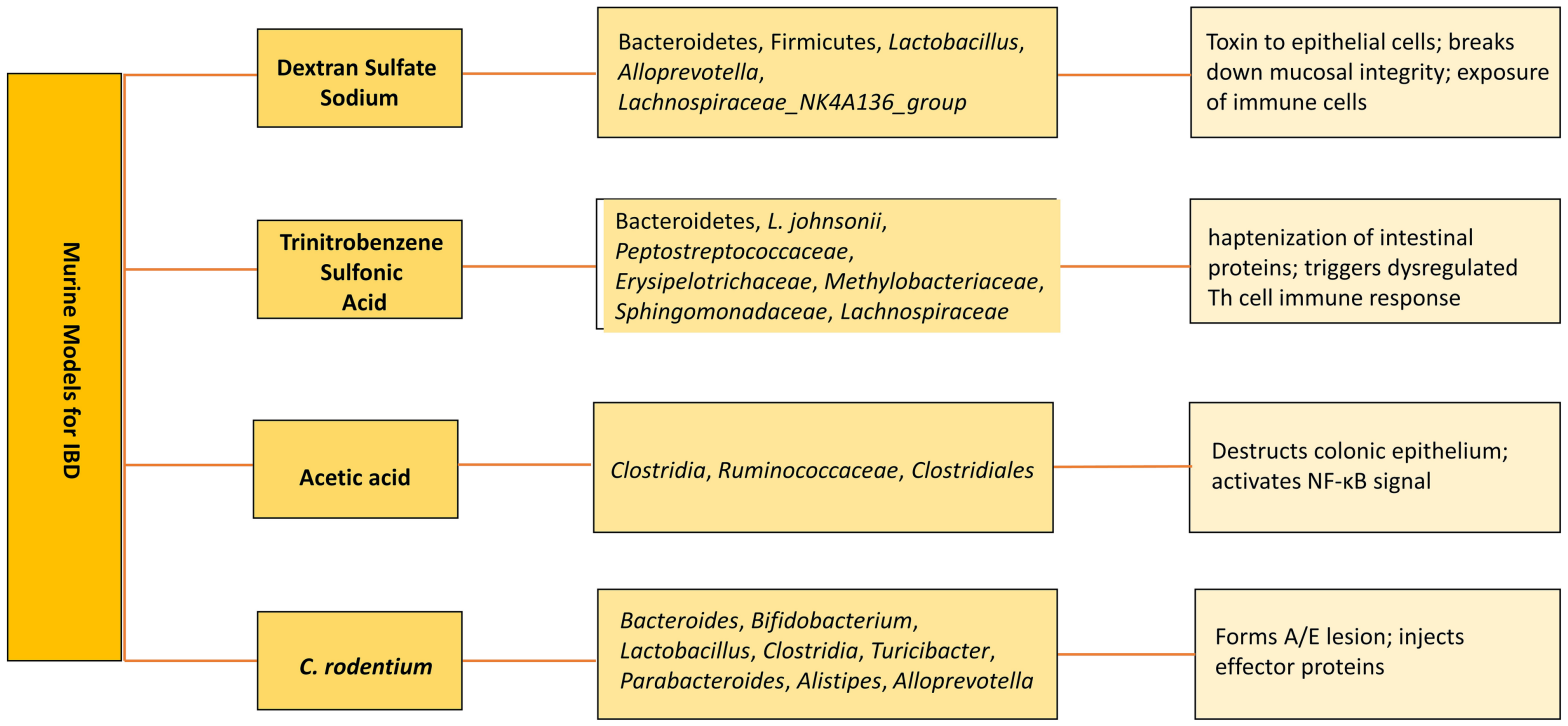
Murine models of IBD. Altered microbiota in various models and subsequent mechanisms to develop IBD.

As an alternative, bEVs have been explored as potential therapies. Various murine models have been developed to study the pathogenesis and therapeutics (Figure 3). For instance, bEVs derived from *C. butyricum* have been shown to reduce colitis in mice induced by DSS by modifying the gut microbiota makeup and repolarizing the M2 macrophages (Liang et al., 2022). Similarly, *L. plantarum* Q7b-derived EVs have been found to improve dysregulated gut microbiota and enhance its diversity in IBD mice (Hao et al., 2021). In addition, bEVs originating from *Akkermansia muciniphila* have been shown to enhance the expression and performance of tight junctions, therefore restoring the intestinal permeability of Caco-2 cells (Chelakkot et al., 2018). *Bifidobacterium longum* EVs demonstrate anti-inflammatory properties by stimulating the release of IL-10 from splenocytes and co-cultures of DC and CD4^+^ T cells. In addition, the protein composition of EVs exhibited a rise in the concentration of extracellular solute-binding proteins, ABC transporters, and proteins involved in quorum sensing. These proteins have been previously demonstrated to play a significant role in the anti-inflammatory action of other strains of *B. longum* (Mandelbaum et al., 2023). *F. prausnitzii-*derived EVs reduced the severity of colitis induced by DSS by influencing intestinal mucosal barrier function and the immune profile (Ye et al., 2023). sRNA52320, discovered in OMVs extracted from *P. aeruginosa*, can decrease the inflammatory response of airway epithelial cells and also inhibit neutrophil activation and infiltration in the lungs of mice (Koeppen et al., 2016). bEVs derived from several microbiota-derived bacteria playing a role in IBD therapeutics are enlisted in Table 3.

**Table 3:** Role of bEVs in IBD therapy.

Bacteria	Model used for clinical trial	Outcome	Reference
*Lactobacillus reuteri*	Broilers	Attenuation of LPS-induced inflammation by improving growth performance, reducing mortality, and alleviating intestinal injury. Suppression of LPS-induced expression of pro-inflammatory genes TNF-*α*, IL-1β, IL-6, IL-17, and IL-8 improved the expression of anti-inflammatory genes (IL-10 and TGF-β) in the jejunum.	Hu et al. (2021)
*Lactobacillus casei*	Caco-2 cell line	Significant increase in IL-10 and IFNγ levels. Level of IL-17A and IFNγ decreased while increasing the concentrations of IL-4 and IL-10	Vargoorani et al. (2020)
*Bacteroides vulgatus*	DSS-induced colitis in mice	Decreased the expression of colonic TNF-a, IL-1b, and IL-6 in DSS-induced mouse colitis. Reduced the secretion of TNF-a, IL-1b, and IL-6 in macrophages stimulated by LPS *in vitro*. Downregulated the expression of Ccl19, Cd19, Cd22, Cd40, and Cxcr5 genes in mice colon.	Liu et al. (2022)
*Lactobacillus plantarum*	DSS-induced colitis in mice	Pro-inflammatory cytokine levels including IL-6, IL-1b, IL-2, and TNF-a were reduced significantly. Pro-inflammatory bacteria (Proteobacteria) were Reduced, and the anti-inflammatory bacteria (Bifidobacteria and Muribaculaceae) were increased	Hao et al. (2021)
*Pediococcus pentosaceus*	1-DSS-induced acute colitis mice model 2-Excisional wound healing model	1-Prevention of colon shortening and loss of crypt architecture 2-Accelerated wound closure through the recruitment of PD-L1 expressing myeloid cells	Alpdundar Bulut et al. (2020)
*Lactobacillus rhamnosus GG*	DSS-induced acute colitis mice model	Pro-inflammatory cytokines (TNF-, IL-1, IL-6, and IL-2) were suppressed effectively. Reshaping the gut microbiota and alteration of metabolic pathways	Tong et al. (2021)
*Clostridium butyricum*	DSS-induced acute colitis mice model	Reduced inflammatory cell infiltration and mucus layer damage in the colon	Ma et al. (2022)
*Clostridium butyricum*	DSS-induced acute colitis mice model	Improved the remission of murine colitis and polarized the transformation of macrophages to the M2 type. Restored dysbiosis	Liang et al. (2022)
*Lactobacillus kefir*, *Lactobacillus kefiranofaciens*, and *Lactobacillus kefirgranum*	Caco-2 cell line and 2,4,6 trinitrobenzene sulfonic acid-induced IBD mouse model	Suppressed the production of inflammatory cytokines in TNF-α-induced inflammation Reduced the level of both mRNA expression and secretion of IL-8	Seo et al. (2018)
*Lactobacillus sakei* subsp. *sakei* NBRC15893	Murine Peyer’s patch cells	Enhanced IgA production by activating host TLR2 signaling through its cell wall components	Yamasaki-Yashiki et al. (2019)

Bioengineered EVs have been found to play a significant role in alleviating IBD. Naturally produced EVs are released by cells autonomously, without any exogenous alterations. They possess intrinsic features, including the capacity to transport proteins, RNAs, and other chemicals that facilitate cellular communication and tissue regeneration. On the other hand, bioengineered EVs are modified either prior to or subsequent to isolation to improve their therapeutic efficacy. These alterations enhance EV stability, bioactivity, targeting, and delivery (de Abreu et al., 2020). Bioengineered bEVs are providing immense potential in the field of IBD therapeutics. Recently, large-scale production of engineered microvesicles derived from the probiotic *L. plantarum* was accomplished, including fucoxanthin in these microvesicles (FX-MVs). The FX-MVs exhibited a 150-fold increase in production and higher protein content compared to the naturally released MVs of the probiotic. In addition, FX-MVs enhanced the gastrointestinal stability of *fucoxanthin* and substantially prevented H_2_O_2_-induced oxidative damage by efficiently scavenging free radicals, thereby significantly reducing the occurrence of colitis (Liang et al., 2023). MVs originating from *Bacteroides thetaiotaomicron* (Bt-MVs) were genetically modified to produce and consistently transport keratinocyte growth factor-2 (KGF-2), a therapeutic protein obtained from humans, into the GIT of mice. This was carried out to provide protection against tissue inflammation and injury. The altered Bt-MVs effectively decreased the severity of the disease and facilitated the healing and restoration of the epithelial tissue in mice with DSS-induced colitis (Carvalho et al., 2019). Nanoprobiotics are probiotics that have been encapsulated or combined with nanotechnology to improve their transport, stability, and bioavailability in the GIT. In contrast to conventional probiotics, which are live bacteria that provide health advantages upon administration, nanoprobiotics employ nanoparticles to shield the probiotics from adverse gastrointestinal environments, enhance their absorption, and potentially enhance their therapeutic efficacy (Durazzo et al., 2020). Nanoprobiotics prepared from EcN-1917 probiotic-derived MVs were encapsulated with manganese dioxide nanozymes. Successful attachment to the inflamed lining of the colon and removal of excessive reactive oxygen species in the intestinal cavity of the mouse model of IBD was observed. Furthermore, utilizing these nanoprobiotics, along with the anti-inflammatory metformin, enhanced the abundance and variety of the gut microbiota and improved the inflammatory condition of the microenvironment (Li et al., 2023).

Till now, there is very limited literature available on the application of microbiota-derived bEVs in relieving IBD. EVs originated from other sources, stem cells, and maternal milk have been well-studied and are being applied for IBD therapeutics. Several challenges hinder the application of bEVs as successful therapeutic targets. These mainly include heterogeneity in size and composition, variability and treatment response among patients, lack of bEV-based murine models to conduct clinical trials, and less specificity to target the inflamed cells. Moreover, the altered microbiota profile of developed murine models does not match the altered microbiota profile of IBD patients. To better understand the pathogenesis and enhance the therapeutic potential, more reliable models are compulsory.
